# Impact of clinician experience on the accuracy of static guided implant placement in post-extraction sockets: a cadaver study

**DOI:** 10.1007/s00784-026-07041-0

**Published:** 2026-07-25

**Authors:** Francesco Pera, Camillo Vocaturo, Veronica Stendardo, Armando Crupi, Marta Bezzi, Umberto Gibello, Andrea Roccuzzo

**Affiliations:** 1 Department of Surgical Sciences - Dental School, University of Turin, Via Nizza 230, Turin, 10126 Italy; 2 DIMEAS, Politecnico di Torino, Corso Duca degli Abruzzi 24, Turin, 10129 Italy; 3 Shanghai Perio-Implant Innovation Center, Institute of Integrated Oral, Craniofacial and Sensory Research, Shanghai Ninth People’s Hospital, Shanghai Jiao Tong University School of Medicine; College of Stomatology, Shanghai Jiao Tong University; National Center for Stomatology; National Clinical Research Center for Oral Diseases; Shanghai Key Laboratory of Stomatology; Shanghai Research Institute of Stomatology, 4F Building 1, 115 Jinzun Road, Pudong Research Campus, Shanghai, 200115 China; 4 Department of Periodontology, School of Dental Medicine, University of Bern, Bern, Switzerland

**Keywords:** Accuracy, Static guided implant placement, Cadaver model, Dental implants

## Abstract

**Objectives:**

To evaluate the accuracy of s-CAIS in immediate post-extraction sockets using a cadaver model and to assess whether operator experience influences implant placement precision and surgical time.

**Methods:**

Twenty teeth (incisors: 9; premolars: 8; molars: 3) were extracted from two fresh defrozen cadaver heads, and implants were planned using a digital workflow integrating cone beam computed tomography and intraoral scanning. Custom-made tooth-supported surgical guides were fabricated and used by two operators: one expert (> 5 years of experience) and one non-expert (< 5 years of experience), both without prior guided surgery experience. Twenty implants were placed. Post-operative scans were taken and superimposed to the pre-operative plans to measure global head deviation, global tip deviation, and angular deviation. Data were stratified by operator experience, jaw location, and implant position.

**Results:**

No significant differences were found between expert and non-expert operators in head deviation (1.26 ± 0.51 mm vs. 1.54 ± 0.63 mm, *p* = 0.29), tip deviation (1.70 ± 0.50 mm vs. 1.81 ± 0.53 mm, *p* = 0.64), angular deviation (3.83 ± 1.90° vs. 3.32 ± 1.69°, *p* = 0.54), or surgical time (3.60 ± 1.45 min vs. 4.31 ± 2.67 min, *p* = 0.84). Anterior implants showed significantly greater head (1.69 ± 0.57 mm vs. 1.13 ± 0.46 mm, *p* = 0.03) and tip deviations (1.99 ± 0.41 mm vs. 1.54 ± 0.50 mm, *p* = 0.04) compared to posterior implants. Mandibular implants required longer surgical time than maxillary implants (4.90 ± 2.37 min vs. 2.87 ± 1.01 min, *p* = 0.03).

**Conclusion:**

Within the limitations of this cadaver study, s-CAIS demonstrated predictable accuracy for immediate implant placement in post-extraction sockets, with operator experience not significantly influencing placement precision or surgical time. Static guided surgery may standardize outcomes and support surgical training in implant dentistry.

**Clinical significance:**

The use of s-CAIS in a cadaver model demonstrated predictable accuracy for immediate implant placement in post-extraction sockets irrespective of operator experience. Its use might be considered for clinical training sessions.

## Introduction

Over the last decades, dental implantology has progressively shifted from conventional delayed placement protocols to immediate protocols aimed at reducing treatment time and preserving peri-implant tissues. Among these, immediate implant placement in post-extraction sockets has gained increasing attention due to its potential to limit hard and soft tissue remodeling and reduce the number of surgical interventions [1, 2]. The purpose of immediate implant placement is to take advantage of the early healing phase to preserve hard and soft tissue volume and to optimize esthetic outcomes, particularly in the anterior maxilla. Clinical studies have reported long-term survival rates ranging from 95% to 99%, with no statistically significant differences compared with delayed implant placement [3, 4].

Despite these favorable outcomes, immediate implant placement remains a technically demanding procedure. The extraction socket represents a dynamic biological environment characterized by early bone remodeling and physiological resorption, particularly of the buccal cortical plate [5, 6]. These dimensional changes, which occur during the first weeks following extraction [7], may compromise implant positioning and primary stability. Furthermore, the irregular morphology of post-extraction sockets and the lack of intact cortical walls increase the risk of angular and linear deviations during implant placement.

Accurate three-dimensional implant positioning is critical to achieve optimal prosthetic outcomes and to avoid biological and mechanical complications. In post-extraction sites, conventional freehand placement may be influenced by the residual socket anatomy, often guiding drills along the axis of the extracted root rather than the prosthetically ideal trajectory. This may result in buccal perforation, prosthetic misalignment, or compromised emergence profiles [8–10].

To improve accuracy and predictability, computer-assisted implant surgery (CAIS) systems have been developed to transfer the virtual preoperative plan to the surgical site, achieving significantly greater accuracy compared with the freehand approach [11, 12]. In static CAIS (s-CAIS), surgical accuracy is enhanced by translating virtual plan into the operative field through stereo lithographically manufactured surgical guides. This digital workflow, which integrates Cone Beam Computed Tomography (CBCT) and digital impression acquisition [13], has been shown to improve implant placement accuracy compared with freehand techniques, particularly in healed ridges [14]. However, s-CAIS is not free from limitations, as errors may be introduced at multiple stages of the workflow, including data acquisition, image superimposition, guide fabrication, and intraoperative guide-fit discrepancies [15].

In addition to anatomical and technical factors, operator experience has traditionally been considered a critical factor influencing surgical outcomes in implantology. Freehand techniques heavily on tactile feedback and spatial perception, often resulting in greater variability among less experienced clinicians. Guided surgery has been proposed to standardize implant placement and potentially reduce the influence of surgical skills. Several studies have reported comparable accuracy between experienced and novice operators when guided techniques are used; however, these investigations have primarily focused on healed ridges or in vitro models, where bone conditions and guide support are optimal [16–18].

Immediate implant placement in post-extraction sockets represents a more challenging clinical scenario. Reduced guide stability, limited tooth support, and compromised cortical anatomy may increase the risk of deviations even when guided protocols are applied. To date, no published studies have specifically evaluated whether operator experience influences the accuracy of static guided implant placement in immediate post-extraction sockets [19]. This lack of evidence limits the understanding of whether s-CAIS can truly compensate for differences in surgical expertise in immediate implant protocols.

The primary objective of this pilot study was to investigate the accuracy of static computer-assisted implant placement in immediate post-extraction sockets in a cadaver experimental model, by matching post-operative digital scans to pre-operative planning data to quantify three-dimensional deviations.

## Materials and methods

This study was aimed to investigate the accuracy of implant placement in post extraction sockets using s-CAIS system, in fresh defrozen cephali. Samples were donated by individuals for scientific purposes and an official statement to handle them was obtained from the local authority prior to the initiation of the study. No specific ethical approval was sought neither obtained since not required by the local legislation. The study was conducted according to the 2018 revised guidelines of the Declaration of Helsinki.

For this study, two males adult Caucasian fresh defrozen cephali, fixed with 10% formalin, were selected. Subjects were partially edentulous in both jaws. An experienced operator (F.P.), with more than 15 years of experience in implant dentistry, carefully evaluated the two samples clinical anatomical conditions and performed extraction of 10 teeth per sample (Total: incisors: 9; premolars: 8; molars: 3) according to the following inclusion criteria:

1. Absence of macroscopic pathological alterations in the maxillary and mandibular bone.
2. Intact soft-tissue surrounding the surgical area.
3. Sufficient residual dental elements (n = > 4) to stabilize a custom-made surgical guide.

Tooth extraction was performed following a minimally invasive approach without performing any osteotomy and/or odontotomy paying attention to maintain intact the buccal bone wall and avoiding root remnants.

The whole prosthetic-driven implant treatment was designed through a digital workflow. Each sample underwent Intraoral Optical Surface Scanning (Aorascan 3; Shining 3D) and pre-operative high- resolution CBCT imaging (NNT—Medical SuiteR) using standardized acquisition parameters (110 kV, 1.94 mA, 3.6 s exposure time, 685.41 DAP (mGy×m2), a field of view (FOV) of 100 × 140 mm, and a voxel size of 0.25 mm).

**Fig. 1:**
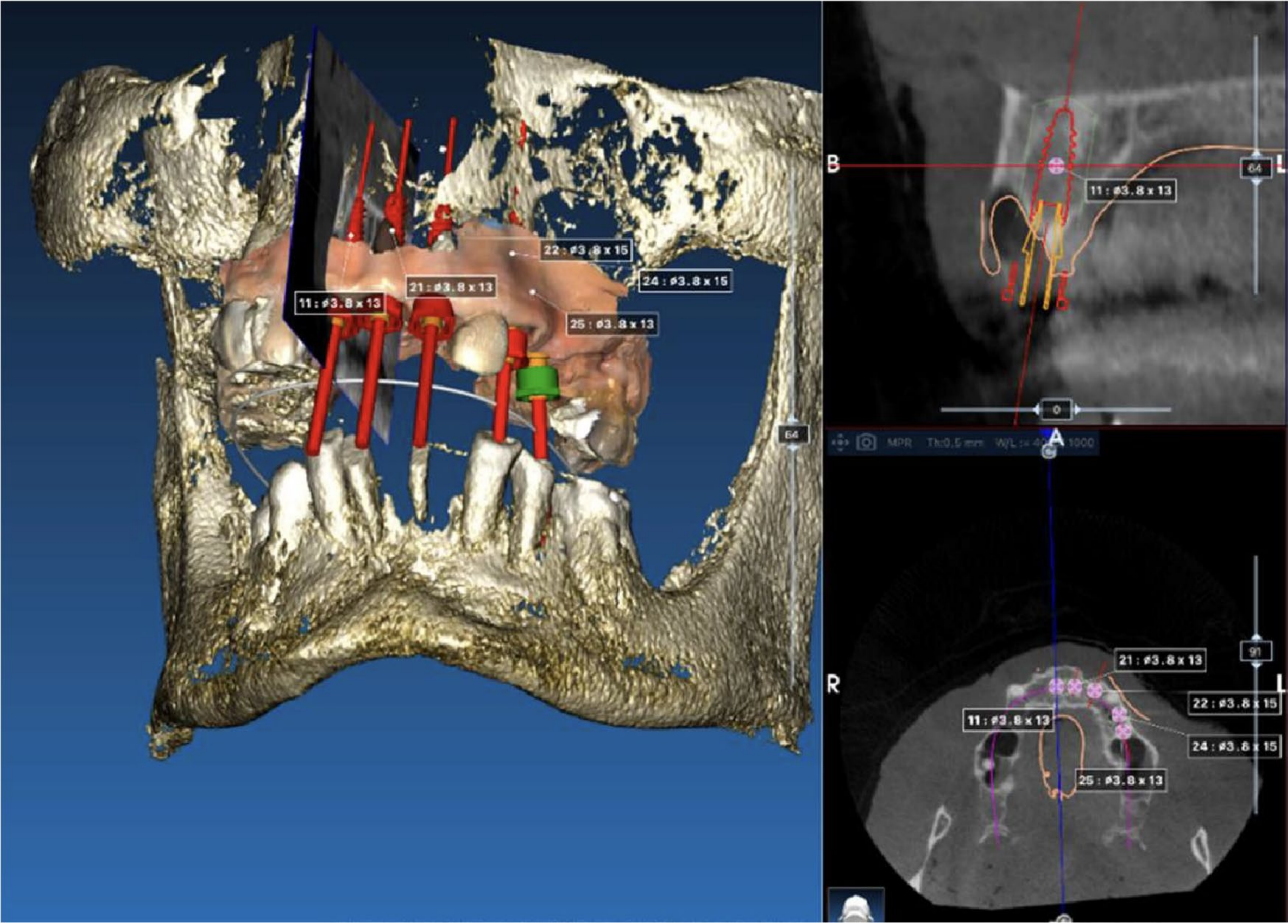
Rapresentative digital scenario of implant placement planning by means of a dedicated software

The digital data from CBCT (DICOM files) and intraoral scanner (STL files) were imported and superimposed using an AI algorithm process within the implant planning software (Real Guide, version 5.0). Implant positions were virtually planned according to prosthetic requirements and anatomical constraints, using the manufacturer’s implant library to ensure correspondence between planned and placed implants (Fig. 1). More specifically, the digital planning was performed following immediate implant placement protocols, inserting all implants 2 mm below the bone crest, anticipating the prevention of the buccal bone wall following surgery [20].

**Fig. 2:**
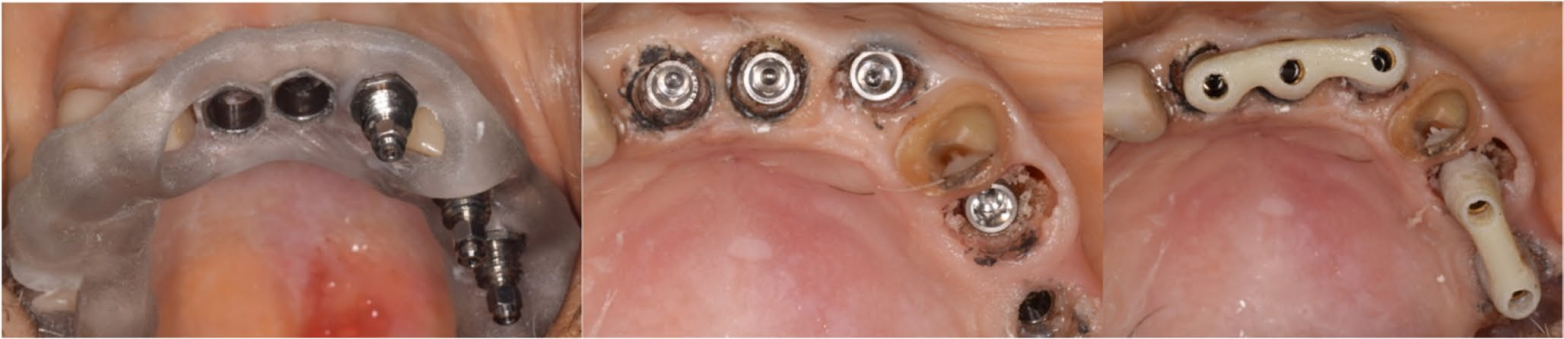
Clinical representative scenario of implants placed within the maxilla with a custom-made tooth-supported surgical guide (**a**). Clinical appearance after removal of the guide (**b**) and after tray-in of the prefabricated provisional fitting test restoration (**c**)

Based on the implant planning, four surgical guides were fabricated, one for each arch (Fig. 2). The guides exhibited standardized features, including a tooth-supported design with an integrated metal sleeve and a flapless surgical approach [21].

The surgical procedure was performed by two operators with different experience in implant dentistry. More specifically, one operator was classified as non-expert (NES; senior oral surgery resident at University of Turin, with less than 5 years of clinical experience), while the second as expert (ES; oral surgeon specialist with more than 5 years of clinical experience in implant dentistry). Both surgeons involved in the study had no prior experience with static guided implant surgery. Before the surgical session, each operator was calibrated by a third senior operator with detailed instructions on how to use the surgical kit and the drilling sequence for each implant type. Each operator performed two surgical procedures (each jaw each). All implants were placed using full-arch tooth-supported surgical guides (Mech & Human) (Fig. 1). Prior to each procedure, the correct fit of every surgical guide was carefully verified to guarantee intraoperative stability and transfer the digital planning to the surgical field. The guide adaptation was verified visually and by gentle finger pressure to confirm complete seating and the absence of any rocking or instability. No guide required minor refinement of the intaglio surface to achieve passive and stable adaptation. All implants were placed using a fully guided protocol according to the manufacturer’s drilling sequence. The operative time was recorded by an external observer, starting when the surgical motor was activated and stopping upon completion of the implant insertion in each site.

**Fig. 3:**
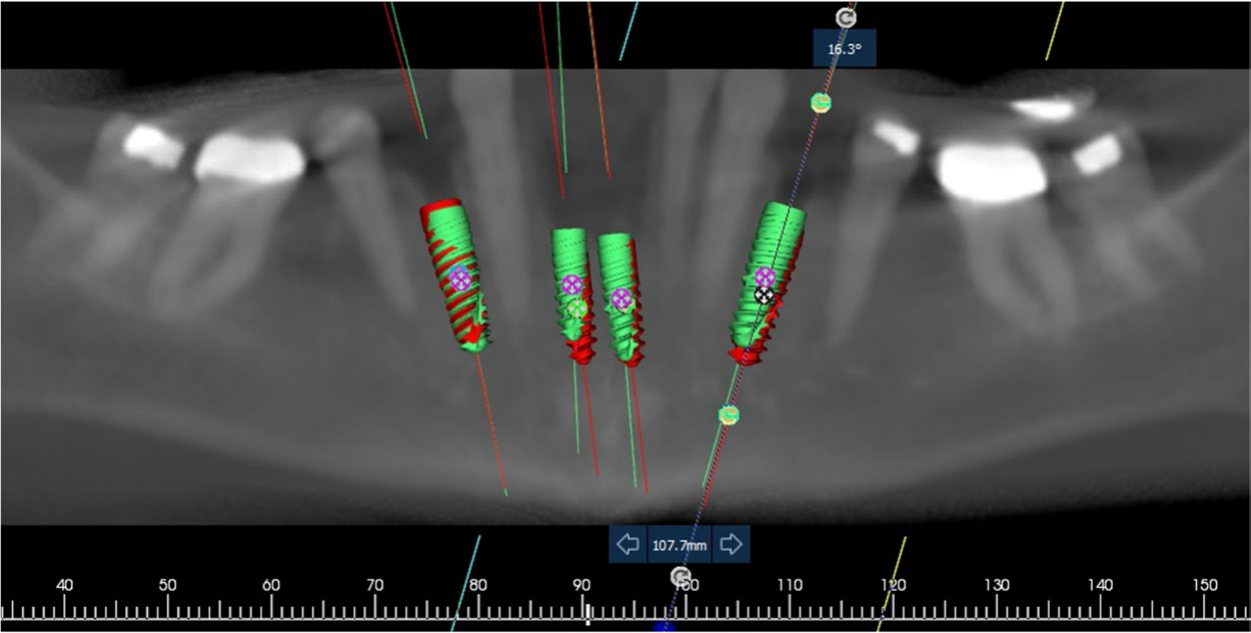
Representative 3D digital scenario showing the planned implants position (green) and the real implants position (red) allowing for discrepancies measurements

**Fig. 4:**
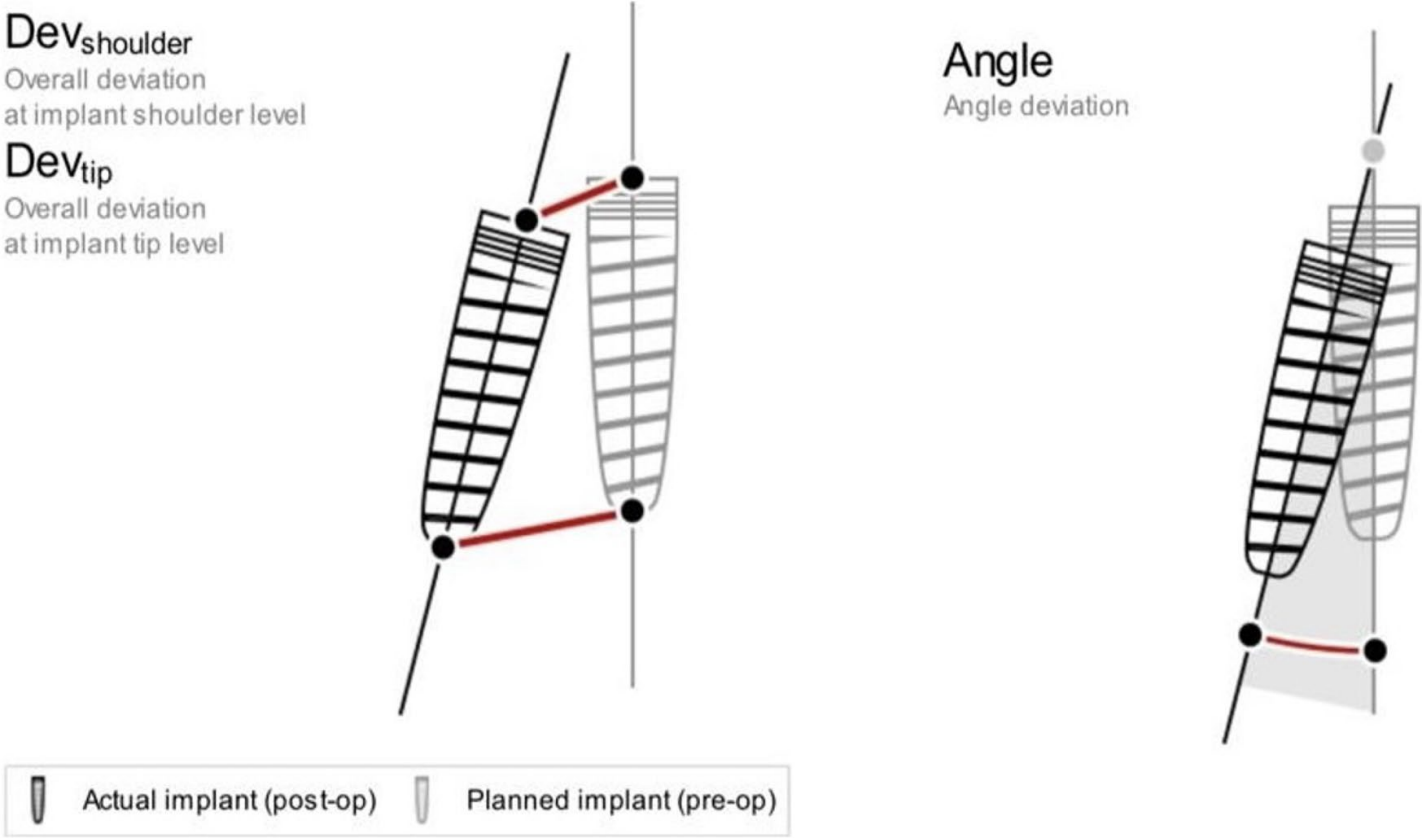
Graphical 2D representation of calculated linear and angular deviations between the planned and real implant positions

After completion of the surgical procedure, the guide was carefully removed and each jaw immediately provisionally restored with a previously fabricated dedicated superstructure. The proper clinical fitting was verified at the end of the surgical procedure according to a previously validated methodology (Fig. 3) [22]. Immediately after the surgical session, a post-operative intraoral optical surface scan (IOS) was performed using dedicated scan body (Mech & Human) to record the implant position. The resulting STL files were imported into the planning software and an experienced dental technician, digitally reconstructed the position of each placed implant based on the scan body geometry according to a previously validated methodology [23]. Consequently, the planned and placed implants were superimposed using the same software (Real Guide, version 5.0), allowing quantitative evaluation of deviations between planned and placed implant positions (Fig. 3). Within the same software, the following bi-dimensional measurements were calculated (Fig. 4):

- Global head deviation (mm), defined as the 3-dimensional distance between the head centroids of the planned and placed implants.
- Global tip deviation (mm), defined as the 3-dimensional distance between the tip of the planned and placed implants.
- Angular deviation (degrees), defined as the angle between the vertical axes of the planned and placed implants.

### Data analysis

Data were reported as mean ± standard deviation (SD). The normality of the distributions was evaluated through Shapiro-Wilk and comparisons between two groups were made by independent sample t-test or Mann–Whitney test depending on the distribution. Data were stratified according to operator experience level (ES vs. NES), jaw (maxilla vs. mandible), and implant position (anterior vs. posterior). A *p* value < 0.05 was considered statistically significant. Analyses were performed with a dedicated software (R; version 4.2.1). The statistical analysis was performed by a professional biostatistician not involved in any part of the study.

## Results

From the originally 20 planned implants, 19 were available for analysis since one implant was excluded due to fracture of the buccal bone wall during implant placement and consequent lack of primary stability. No additional intra-surgical complications were detected. All implants were of the same brand (Shard model, Mech & Human) and had specific diameter and length (3.25 × 13 mm, 3.25 × 11.5 mm, 4 × 15 mm, and 4 × 13 mm). Implants were equally distributed with respect to the jaw and to operator experience. Mean deviations at the implant head (1.26 ± 0.51 mm vs. 1.54 ± 0.63 mm, *p* = 0.29), tip (1.70 ± 0.50 mm vs. 1.81 ± 0.53 mm, *p* = 0.64), angular deviation (3.83 ± 1.90° vs. 3.32 ± 1.69°, *p* = 0.54), and surgical time (3.60 ± 1.45 min vs. 4.31 ± 2.67 min, *p* = 0.84) were comparable between groups. When comparing implant location, mandibular implants (*n* = 10) required significantly longer surgical time than maxillary implants (*n* = 9) (4.90 ± 2.37 min vs. 2.87 ± 1.01 min, *p* = 0.03), though positional accuracy parameters were similar between sites (*p* > 0.05). Regarding anterior vs. posterior positioning, anterior implants (*n* = 9) demonstrated significantly greater deviation at both the head (1.69 ± 0.57 mm vs. 1.13 ± 0.46 mm, *p* = 0.03) and tip (1.99 ± 0.41 mm vs. 1.54 ± 0.50 mm, *p* = 0.04) compared to posterior implants (*n* = 10), while angular deviation and surgical time were comparable (*p* > 0.05).

The analysis of 19 dental implants revealed no significant differences in placement accuracy or surgical time between expert (*n* = 10) and non-expert (*n* = 9) surgeons. All reconstructions showed clinical signs of optimal fitting. Details of all measurements are provided in Tables 1, 2 and 3.

**Table 1:** Overall evaluated differences between the expert (ES) (*n* = 10 implants) and non-expert (NES) (*n* = 9 implants) surgeon group

Group	*N* (implants)		Deviation head (mm)	Deviation tip (mm)	Angular deviation (degrees)	Time (minutes)
ES	10	Mean	1.26	1.70	3.83	3.60
		SD	0.51	0.50	1.90	1.45
NES	9	Mean	1.54	1.81	3.32	4.31
		SD	0.63	0.53	1.69	2.67
*p-*value			0.29	0.64	0.54	0.84

*SD* Standard Deviation

**Table 2:** Overall evaluated differences with respect to implant location (maxilla vs. mandible)

Site	*N* (implants)		Deviation head (mm)	Deviation tip (mm)	Angular deviation (degrees)	Time (minutes)
Maxilla	9	Mean	1.47	1.69	3.70	2.87
		SD	0.64	0.53	2.31	1.01
Mandible	10	Mean	1.33	1.81	3.49	4.90
		SD	0.54	0.50	1.23	2.37
*p*-value			0.61	0.61	0.81	0.03*

*SD* Standard Deviation

*Statistically significant difference

**Table 3:** Overall evaluated differences with respect to implant location (anterior vs. posterior)

Position	*N* (implants)		Deviation head (mm)	Deviation tip (mm)	Angular deviation (degrees)	Time (minutes)
Anterior	9	Mean	1.69	1.99	3.96	4.34
		SD	0.57	0.41	1.88	2.56
Posterior	10	Mean	1.13	1.54	3.26	4.34
		SD	0.46	0.50	1.70	2.56
*p*-value			0.03*	0.04*	0.40	0.66

*SD* Standard Deviation

*Statistically significant difference

## Discussion

The objective of this study was to preliminary explore the accuracy of static computer-assisted implant surgery (s-CAIS) in post-extraction sockets and to evaluate whether operator experience influences implant placement accuracy and operative time in a cadaver model. The obtained results demonstrated that s-CAIS was satisfactory in terms of geometric precision (i.e., angular and linear deviations), with negligible differences between experienced and non-experienced operators.

One of the main objectives of this study was to evaluate the role of operator experience. Traditionally, implant placement accuracy has been linked to surgical expertise, particularly in freehand procedures. Guided surgery has been proposed as a tool to standardize implant placement and potentially reduce operator-dependent variability. The present results support this concept, as no significant differences in surgical accuracy or time were found between operators with different degrees of experience; s-CAIS effectively minimizes the influence of operator skills, facilitating precise implant placement even for less experienced clinicians. Several studies have similarly reported no significant differences between novice and expert operators using guided techniques, whereas freehand approaches showed markedly greater deviations among beginners [16, 18]. Also, Reiff et al. confirmed that guided systems reduce variability related to operator experience, highlighting their educational potential [17]. Collectively, these findings suggest that s-CAIS enhances procedural reproducibility, shortens the learning curve and represents an effective educational tool in implant training.

As highlighted by D’haese et al. [15] and Zhou et al. [24] surgical accuracy is not only due to the operator’s manual skills, but also to his ability to manage the digital workflow (i.e., reading the CBCT correctly, aligning the scans, and evaluating the stability of the guide). This may explain why guided surgery has been proposed as a teaching tool capable of reducing the learning curve and supporting less experienced clinicians in achieving predictable outcomes [25].

The overall mean deviations recorded in the present study are consistent with those reported in the literature for static guided implant placement. Tahmaseb et al., in their meta-analysis, reported mean linear deviations of 1.2 mm at the entry point and 1.4 mm at the apex, with a mean angular deviation of 3.5°, which are commonly accepted reference values for s-CAIS accuracy [12]. The slightly higher values obtained in the present study are coherent with recent investigations focusing on the biomechanical specificities of the immediate post-extraction sockets. Chen et al. demonstrated that implant in fresh extraction sockets shows significantly greater linear and angular discrepancies than in healed sites, although remaining within clinically acceptable limits [8]. When data were analyzed by anatomical site, surgical time was significantly longer in the mandible (4.9 min) than in the maxilla (2.87 min), this could be advocated to the limited intraoral access to the posterior sites and higher bone density of the mandible.

Larger linear deviations were observed in the anterior regions when stratified by position. This finding may be related to anatomical and technical factors, including thinner cortical bone, reduced guide support, and limited stabilization of tooth-supported guides in the anterior area. Chen et al. [8] similarly reported greater discrepancies in post-extraction anterior sites, while Nguyen et al. demonstrated that guides supported by fewer anterior teeth resulted in significantly higher apical and angular deviations during immediate implant placement in the maxillary central incisor region [26]. These results demonstrate that post-extraction anterior sites are a challenge for the clinician, where guide stabilization and cortical integrity are critical to obtain surgical precision. More specifically, when focusing on the magnitude of apical deviation (up to nearly 2 mm), it should be underlined how this inaccuracy was likely caused by the drill ‘skating’ on the sloping walls of the socket. From a clinical perspective, such area does not allow any margin for error to minimize the risk of peri-implant buccal bone resorption [27–29] and late development of peri-implant soft-tissue dehiscences [30] which have a major aesthetic impact [31]. Conversely, posterior sites often benefit from wider dental support and greater guide stability, which can help improve accuracy. Angular deviation did not differ significantly between the anterior and posterior regions, suggesting that linear deviations were more sensitive to driving stability than implant angle.

No significant differences in accuracy were observed between maxillary vs. mandibular sites. However, surgical time was significantly longer in the mandible. This may be attributed to increased bone density, limited accessibility, and mouth opening in posterior mandibular regions, even in a cadaver model.

From a clinical perspective, the deviations observed in this study remain within the range considered acceptable for functional and esthetic implant success. The immediate provisional restoration tested after surgery provided a qualitative clinical validation of the achieved accuracy, as satisfactory fitting was observed in all cases. This finding indicates that the measured deviations were compatible with prosthetically acceptable implant positioning. As suggested by Sadilina et al., who emphasized that the true value of computer-assisted implant surgery extends beyond numerical accuracy, encompassing its ability to ensure biologically and prosthetically sound outcomes. The favorable clinical adaptation of the provisionals observed in this study thus supports the translational validity of the obtained results [32].

Despite the encouraging findings, some limitations should be acknowledged. The study was conducted on cadaver samples, which do not fully reproduce intraoral conditions such as soft-tissue resistance or patient-related variables. The limited sample size in terms of placed implants and the use of a single guide design also restrict generalization. It is within this context that, larger studies should be performed to verify if the detected observed 0.3 mm trend in operator-dependent deviation becomes statistically significant, thereby refining the definition of ‘operator sensitivity’ in guided surgery. Nevertheless, the standardized setup allowed precise control of variables and provided valuable baseline data for future clinical studies. Moreover, it should be recalled that the reported buccal bone fracture serves as a vital reminder that a surgical guide is no substitute for clinical judgment or tactile feedback. Blind reliance on a guide that does not incorporate ‘biological backward planning’ may result in a correct implant placement according to the planning software but incorrect implant position to anticipate post-surgical physiological peri-implant bone resorption [33].

Further studies should confirm these results in vivo, comparing static and dynamic navigation in post-extraction sockets and evaluating the influence of different guide designs or sleeve-to-drill tolerances and bone density. This will help to determine the most efficient workflow to maximize immediate implant placement precision.

## Conclusion

s-CAIS provides predictable accuracy in immediate post-extraction sockets in a cadaver model and operator’s experience seemed not to significantly influence it. The use of a custom-made guide appears a reliable tool promoting safe implant placement in challenges scenarios. Within the limitations of the implemented model, s-CAIS represents a reliable tool for supporting surgical training in implant dentistry.

## Data Availability

The data that support the findings of this study are available upon reasonable request from the corresponding author. The data are not publicly available due to privacy or ethical restrictions.
